# Supplementary material for: Discovering two general characteristic times of transient responses in solid oxide cells
Source: Nat Commun. 2024 May 30;15:4587. doi: 10.1038/s41467-024-48785-1 (PMC11137161; doi:10.1038/s41467-024-48785-1)
Supplement: Supplementary file 1 — Supplementary Information [file 41467_2024_48785_MOESM1_ESM.pdf]

SUPPLEMENTARY INFORMATION

Discovering two general characteristic times of transient responses in solid oxide cells

Zhaojian Liang[1], Jingyi Wang[2], Keda Ren[2], Zhenjun Jiao[2], Meng Ni[3], Liang An[1], Yang Wang[3], Jinbin Yang[2], and Mengying Li*[1]

[1]Department of Mechanical Engineering & Research Institute for Smart Energy, The Hong Kong Polytechnic University, Hong Kong SAR

[2]School of Science, Harbin Institute of Technology, Shenzhen, 518055, China

[3]Department of Building and Real Estate, The Hong Kong Polytechnic University, Hong Kong SAR

1 Supplementary Methods

Derivation of characteristic times

This section presents the theoretical derivation of the characteristic times for heat and mass transfer. The SOC system can be represented as a control volume with volume $\mathcal{V}$, density $\bar{\rho}$, and enthalpy $\mathcal{H}$, as illustrated in Supplementary Fig. 1.

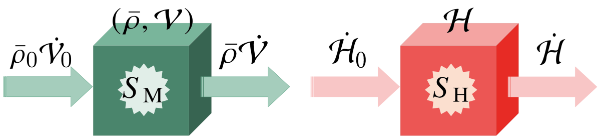

Supplementary Figure 1: Mass and energy conservation of a control volume.

*email: mengying.li@polyu.edu.hk

Mass flux and enthalpy flux across the system boundaries are denoted as $\bar{\rho}\dot{\mathcal{V}}$ and $\dot{\mathcal{H}}$, respectively. Within the control volume, electrochemical reactions serve as mass source S_{M} and heat source S_{H}. The temporal changes in mass and heat within the system are influenced by these sources, as well as the associated mass and heat fluxes. The mass conservation of the control volume is articulated in Supplementary Eqs. (1) and (2).

$$\frac{\partial \bar{\rho}}{\partial t}\mathcal{V} = \bar{\rho}_0\dot{\mathcal{V}}_0 - \bar{\rho}\dot{\mathcal{V}} + S_{\mathrm{M}} \tag{1}$$

$$\frac{\partial \mathcal{H}}{\partial t} = \dot{\mathcal{H}}_0 - \dot{\mathcal{H}} + S_{\mathrm{H}} \tag{2}$$

where $\bar{\rho}_0$, $\dot{\mathcal{V}}_0$, and $\dot{\mathcal{H}}_0$ denote the density, volumetric flow rate, and enthalpy flow rate at the inlet. $\mathcal{H}_0$ is the reference enthalpy calculated according to inlet conditions.

In SOCs, the magnitude of S_{M} is constrained by the current and the flow rate of reactants at the inlet. S_{M} can be expressed as a function of the inlet volumetric flow rate, $\dot{\mathcal{V}}_0$, as illustrated in Supplementary Eq. (3),

$$S_{\mathrm{M}} = \mathcal{U}_{\mathrm{R}}\varphi_{\mathrm{R}}\left(\bar{\rho}_{\mathrm{P}} - \bar{\rho}_{\mathrm{R}}\right)\dot{\mathcal{V}}_0 \tag{3}$$

where, $\bar{\rho}_{\mathrm{R}}$ and $\bar{\rho}_{\mathrm{P}}$ denote the densities of reactants and products, respectively. φ_{R} represents the volume fraction of reactants in the inlet flow, with a value ranging from 0 to 1. Additionally, $\mathcal{U}_{\mathrm{R}}$ signifies the utilization rate of the reactant, which also varies between 0 and 1. A utilization rate of $\mathcal{U}_{\mathrm{R}} = 1$ implies that all the reactants are converted into products through electrochemical reactions.

Similarly, the magnitude of S_{H} is also constrained in SOCs. We can establish an empirical relationship between S_{H} and the inlet enthalpy flux, $\dot{\mathcal{H}}_0$, as shown in Supplementary Eq. (4),

$$S_{\mathrm{H}} = a\dot{\mathcal{H}}_0 \tag{4}$$

where, a represents a constant whose order of magnitude is either equal to or less than 1 for typical SOC operations.

Combining Supplementary Eqs. (1) to (4), as well as the scaling rules Supplementary Eq. (5), we can acquire the dimensionless forms of mass and energy conservation equations, as shown in Supplementary Eqs. (6) and (7).

$$t^* = \frac{t}{\tau_{\mathrm{m}}} \text{or} \frac{t}{\tau_{\mathrm{h}}}, \bar{\rho}^* = \frac{\bar{\rho}}{\bar{\rho}_0}, \dot{\mathcal{V}}^* = \frac{\dot{\mathcal{V}}}{\dot{\mathcal{V}}_0}, \mathcal{H}^* = \frac{\mathcal{H}}{\mathcal{H}_0}, \dot{\mathcal{H}}^* = \frac{\dot{\mathcal{H}}}{\dot{\mathcal{H}}_0} \tag{5}$$

$$\frac{\partial \bar{\rho}^*}{\partial t^*} = \frac{\boldsymbol{\tau_m}}{\boldsymbol{\mathcal{V}/\dot{\mathcal{V}}_0}} \left(1 - \bar{\rho}^* \dot{\mathcal{V}}^* + \mathcal{U}_R \varphi_R \frac{\bar{\rho}_P - \bar{\rho}_R}{\bar{\rho}_0}\right) \tag{6}$$

$$\frac{\partial \mathcal{H}^*}{\partial t^*} = \frac{\boldsymbol{\tau_h}}{\boldsymbol{\mathcal{H}_0/\dot{\mathcal{H}}_0}} \left(1 - \dot{\mathcal{H}}^* + a\right) \tag{7}$$

From Supplementary Eqs. (6) and (7), we deduce that $\mathcal{V}/\dot{\mathcal{V}}_0$ and $\mathcal{H}_0/\dot{\mathcal{H}}_0$ can be chosen as the time scales to characterize the transient mass and heat transfer in SOC systems. By defining,

$$\tau_m = \mathcal{V}/\dot{\mathcal{V}}_0 \tag{8}$$

$$\tau_h = \mathcal{H}_0/\dot{\mathcal{H}}_0 \tag{9}$$

Supplementary Eqs. (6) and (7) can be reformulated as,

$$\frac{\partial \bar{\rho}^*}{\partial t^*} = 1 - \bar{\rho}^* \dot{\mathcal{V}}^* + \mathcal{U}_R \varphi_R \frac{\bar{\rho}_P - \bar{\rho}_R}{\bar{\rho}_0} \tag{10}$$

$$\frac{\partial \mathcal{H}^*}{\partial t^*} = 1 - \dot{\mathcal{H}}^* + a \tag{11}$$

It should be noted that $\partial\bar{\rho}^*/\partial t^*$ and $\partial\mathcal{H}^*/\partial t^*$ are of the order of 1 in Supplementary Eqs. (10) and (11). This demonstrates the effectiveness of $\mathcal{V}/\dot{\mathcal{V}}_0$ and $\mathcal{H}_0/\dot{\mathcal{H}}_0$ in representing the time scales of mass and heat transfer in SOC systems.

Analysis Strategy of the thermal responses of SOC

This section explains the strategy applied to investigate the thermal responses of SOC. The total heat source in a planar SOC [1] can be described by Supplementary Eq. (12),

$$S_H^{total} = S_{Ohm}^{Int} + S_{Ohm}^{ADL} + S_{Ohm}^{CDL} + S_{Ohm}^{E} + (S_{irrev} + S_{rev} + S_{Ohm})^{AFL} + (S_{irrev} + S_{rev} + S_{Ohm})^{CFL} \tag{12}$$

where, the Ohmic and irreversible heat sources are positive, and the reversible heat sources are negative for SOEC and positive for SOFC. These heat sources are functions of current and voltage, as illustrated in our previous work [1]. For unique cases such as under open circuit voltage (OCV) or thermoneutral voltage (TNV), the total heat source is null, *i.e.*, $S_H^{total} = 0$. At OCV, the current is null resulting in zero heat sources. At the TNV of SOEC, the negative S_{rev} offsets the positive heat sources. The thermoneutrality at OCV and TNV exemplifies that SOCs at different electrical

states may present the same thermal state.

Supplementary Figs. 2a-b display the transient responses of two identical SOECs, initially set at 1.1 V, then following rapid voltage changes to OCV (≈ 0.9 V) and TNV (≈ 1.35 V) respectively. Despite the identical initial and final thermal states of both SOECs, their temperature relaxation processes differ due to the distinct responses of current and heat source. This example underscores the intricate relationship among heat source, current, and voltage. It suggests that simply adjusting the electrical state does not provide a straightforward method for conducting controlled thermal investigations of SOCs. The variation in the heat source is, in fact, the primary factor influencing temperature change. Therefore, to accurately define the characteristic time of thermal response, we propose altering the heat source rather than the electrical conditions during our thermal investigation.

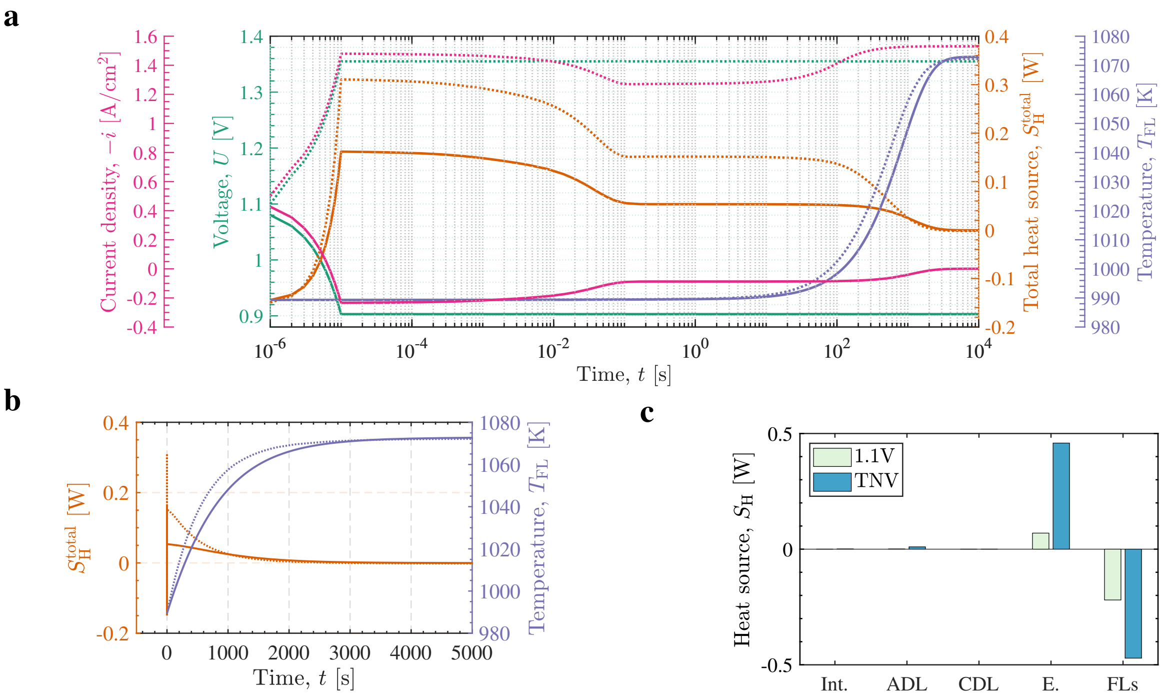

Supplementary Figure 2: Comparison of transient responses of two identical SOECs subjected to rapid voltage variations. The solid lines represent the response when voltage changes from 1.1 V to OCV, and the dashed lines represent the response when voltage transitions from 1.1 V to TNV. Both SOECs adhere to identical structure and boundary conditions, same as the base case (see Table 1 in main text). **a** Responses in logarithmic timeline. **b** Responses in the linear timeline. **c** Comparison of heat sources in different elements at 1.1 V and TNV.

Furthermore, as demonstrated in Supplementary Fig. 2a-b, S_H^{total} undergoes a significant change in the initial 0.1 s, followed by a slower rate of variation over the subsequent few thousand seconds. This pattern bears a resemblance to a step change, justifying the manipulation of the heat source in the SOC thermal investigation. The step change of S_H^{total} provides a reasonable approximation to the actual response of the heat source after electrical variation.

Supplementary Fig.2c provides a comparative analysis of the heat sources within various SOC elements at 1.1 V and TNV. Notably, the dominant heat sources or sinks are found within the functional layers (FLs) and the electrolyte, while the contributions from other elements are relatively insignificant. Therefore, our thermal investigation only considers the heat sources in FLs and electrolyte.

Additionally, it is important to note that the AFL, electrolyte, and CFL are adjacent and exhibit minimal thicknesses, approximately 0.01 mm. Consequently, the temperatures across the AFL, electrolyte, and CFL are nearly uniform in the thickness direction. This uniformity allows for the thermal effects of AFL, CFL, and electrolyte to be approximated as a lumped heat source from a thin region. As such, we model the different thermal states of SOCs by adjusting the total heat source on FLs and electrolyte.

2 Supplementary Results

Validation with experiment

Experiments of transient responses of an SOEC cell were conducted to validate the proposed characteristic time of mass transfer, focusing on the comparison of distinct voltage relaxation processes after the rapid ramp-down of current with different fuel flow rates. The experimental apparatus shown in Supplementary Fig. 3a includes a gas supply system, a furnace, an electrochemical workstation, and an anode-supporting SOFC unit consisting of electrode lead wires and a commercial PEN (Positive Electrode/Electrolyte/Negative Electrode) provided by SOFCMAN Energy. The PEN, with an active area of 16 cm^2, is sealed in a ceramic clamp with the air electrode exposed to air. The fuel gas is supplied at a constant volumetric flow rate $\dot{\mathcal{V}}_{\mathrm{st.}}^{\mathrm{fuel}}$ under standard conditions ($T_{\mathrm{st.}} = 298.15$ K, $p_{\mathrm{st.}} = 1$ atm), and is preheated to about 433 K before flowing into the fuel cavity of the SOFC unit. Preheating is facilitated by a heating band wrapped around the metal tube before entrancing the gas inlet. Oxygen is supplied by an air flow introduced directly onto the air electrode, maintaining a constant flow rate of 500 sccm. The SOFC was operated in a furnace that maintained a consistent operating temperature of 1073.15 K.

During the experiments, the current of SOFC was ramped down to 0 within 1 ms, and the resulting current and voltage were recorded at 0.1 ms intervals by the electrochemical workstation. The details of the experimental parameters and the estimated characteristic times are given in Supplementary Table 1. Supplementary Fig. 3b shows that the current ramps induce the voltage variations, with the interesting observation that these voltage variations continue even beyond the cessation of the current ramps. The voltage responses after the current change are plotted against time in a logarithmic scale as shown Supplementary Fig. 3c. After normalizing the voltage and scaling the time with $\tau_{\mathrm{m,fuel}}$, it

93 is clear that the relaxation time of voltage responses is about $2\tau_{\mathrm{m,fuel}}$. The result further validates the effectiveness of $\tau_{\mathrm{m,fuel}}$ in characterising the mass-transfer process in SOCs.

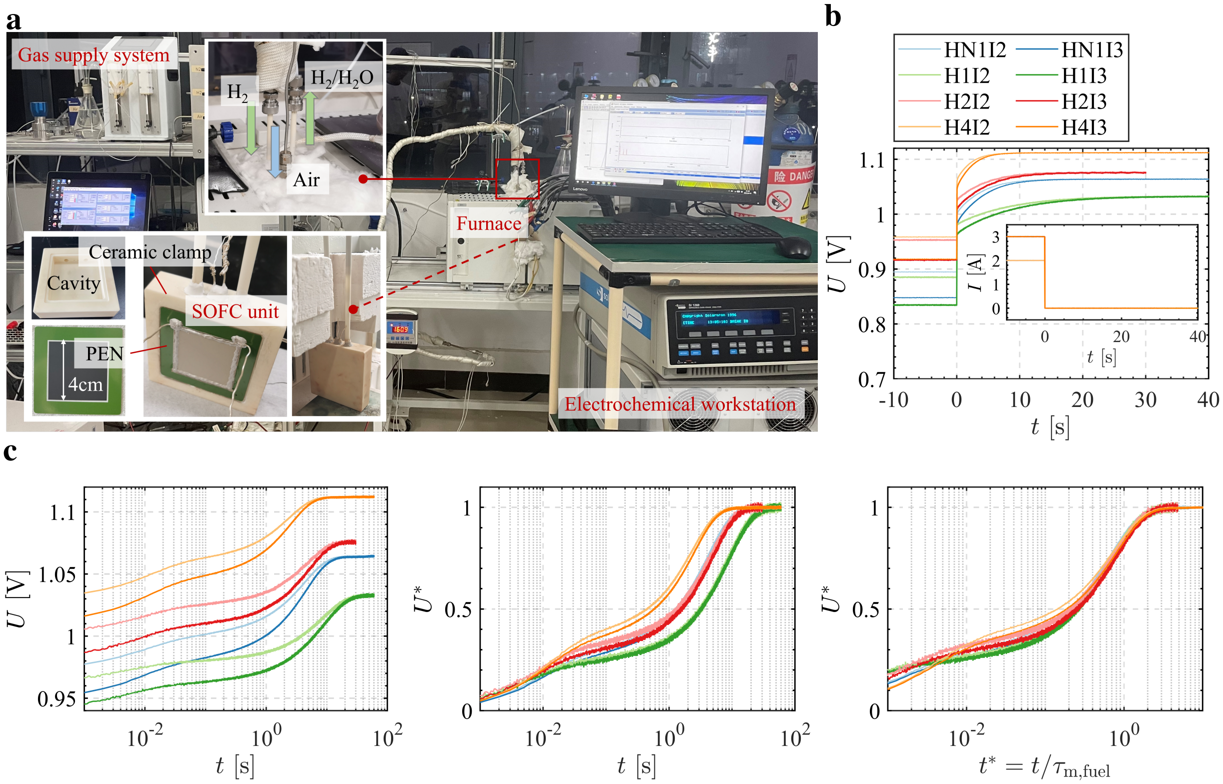

Supplementary Figure 3: SOFC experiment. **a** Experimental setup. **b** The raw response data of voltage and current. The current ramp-down starts at 0 s and finishes at about 0.1 ms. **c** The voltage responses to step changes of current. The voltage is scaled by, $U^{*} = (U - U_{t\approx 0.1\,\mathrm{ms}})/(U_{t\to\infty} - U_{t\approx 0.1\,\mathrm{ms}})$.

94

95 Validation with literature data

96 Supplementary Table 2 details the calculations of τ_{m} and τ_{h}.

Supplementary Table 1: Experimental conditions and parameters of SOFC.

Name	Case	$\dot{\mathcal{V}}_{\text{st.}}^{\text{fuel}}$ [cm^3/min]	$I_{t=0}$ [A][a]	Estimated $\dot{\mathcal{V}}_{\text{in}}^{\text{fuel}}$ [m^3/s][b]	Estimated $\tau_{\text{m}}^{\text{fuel}}$ [s][c]
HN1I2	1	H_2: 100; N_2: 100	2	4.84E-06	6.22
HN1I3	2	H_2: 100	3	4.84E-06	6.22
H1I2	3	H_2: 100	2	2.42E-06	12.45
H1I3	4	H_2: 100	3	2.42E-06	12.45
H2I2	5	H_2: 200	2	4.84E-06	6.22
H2I3	6	H_2: 200	3	4.84E-06	6.22
H4I2	7	H_2: 400	2	9.69E-06	3.11
H4I3	8	H_2: 400	3	9.69E-06	3.11

[a] Each case undergoes a step change from $I_{t=0}$ to 0 A within 1 ms.

[b] $\dot{\mathcal{V}}_{\text{in}}$ denotes the fuel flow rate at the inlet of the fuel cavity, which is estimated by: $\dot{\mathcal{V}}_{\text{in}} = 1.667 \times 10^{-8} \times \dot{\mathcal{V}}_{\text{st.}} \times \frac{T_{\text{preheat}}}{T_{\text{st.}}}$

[c] $\tau_{\text{m}}^{\text{fuel}} \approx \left(L_{\text{cavity}} W_{\text{cavity}} H_{\text{cavity}} + L_{\text{cell}} W_{\text{cell}} \varepsilon_{\text{DL}}^{\text{fuel}} \delta_{\text{DL}}^{\text{fuel}}\right) / \dot{\mathcal{V}}_{\text{in}}^{\text{fuel}} = (0.05 \times 0.05 \times 0.012 + 0.04 \times 0.04 \times 0.22 \times 0.4) / \dot{\mathcal{V}}_{\text{in}}^{\text{fuel}}$

Supplementary Table 2: Comparison of calculated characteristic times and reported relaxation times for cells in response to rapid voltage/current changes.

Cell type	Response variables	Reported relaxation time [s]	Calculated τ_m, τ_h [s]	Remarks (unless specified, the parameters necessary for calculations are sourced from the respective referenced papers.)
3-D planar SOFC [2]	Current	≈ 0.6 [a]	$\tau_m^{fuel} = 0.46$	$\tau_m^{fuel} = (L_{cell} W_{ch} H_{ch} + \varepsilon_{DL}^{fuel} L_{cell} W_{cell} \delta_{DL}^{fuel} + \varepsilon_{foam}^{fuel} L_{cell} W_{cell} \delta_{foam}^{fuel}) / (W_{ch} H_{ch} V_{in}^{fuel})$<br>The fuel side of the planar cell includes channel, Ni foam, DL and FL. However, due to the relatively small volume of the FL, it is disregarded in the calculation.
2-D tubular SOEC [3] (co-electrolysis)	H_2O concentration<br>Temperature	0.255 – 1 [b]<br>515 – 2000 [b]	$\tau_m^{fuel} = 0.42$<br>$\tau_h = 2076$	$\tau_m^{fuel} = L_{cell} \left[(r_{ch}^{outer})^2 - (r_{ch}^{inner})^2 + \varepsilon_{DL}^{fuel} \left((r_{ch}^{outer} + \delta_{cathode}^{fuel})^2 - (r_{ch}^{outer})^2 \right) \right] / \left(V_{in} r_{inject}^2 \right)$<br>$\tau_h \approx \left[(mc_p)_{inject}^{solid} + (mc_p)_{E}^{solid} + (mc_p)_{cathode}^{eff} + (mc_p)_{anode}^{eff} \right] / (\dot{m} c_p)_{in}^{fuel}$<br>In the tubular cell, both the fuel channel and DL adopt a cylindrical shell shape. Heat capacities of gases are neglected due to their relatively small values.
3-D planar SOFC [4]	Fuel utilization<br>Air utilization	≈ 0.7 [a]<br>≈ 0.05 [a]	$\tau_m^{fuel} = 0.49$<br>$\tau_m^{air} = 0.04$	$\tau_m^{fuel} = (L_{cell} W_{ch} H_{ch} + \varepsilon_{DL}^{fuel} L_{cell} W_{cell} \delta_{DL}^{fuel}) / (W_{ch} H_{ch} V_{in}^{fuel})$<br>$\tau_m^{air} = (L_{cell} W_{ch} H_{ch}) / (W_{ch} H_{ch} V_{in}^{air})$<br>The FLs are neglected in calculation due to their relatively small volumes.
Quasi 2-D SOEC stack [5] (co-electrolysis, 40 cells)	Current<br>Stack temperature	< 1 [b]<br>> 900 [b]	$\tau_m^{fuel} = 0.23$<br>$\tau_h = 3100$	$\tau_m^{fuel} = (L_{cell} W_{ch} H_{ch} + \varepsilon_{DL}^{fuel} L_{cell} W_{cell} \delta_{DL}^{fuel}) / (W_{ch} H_{ch} V_{in}^{fuel})$.<br>$\tau_h \approx \left[(mc_p)_{int}^{solid} + (mc_p)_{E}^{solid} + (mc_p)_{cathode}^{eff} + (mc_p)_{anode}^{eff} \right] / \left[(\dot{m} c_p)_{in}^{fuel} + (\dot{m} c_p)_{in}^{air} \right]$<br>Heat capacities of gases are neglected due to their relatively small values.
Flat-tube SOC [6] (CO_2 electrolysis)	Electrical impedance	≈ 1.0 [c]	$\tau_m^{fuel} = 0.97$	$\tau_m^{fuel} = (\mathcal{V}_{ch} + \varepsilon_{DL}^{fuel} \mathcal{V}_{DL}^{fuel} + \varepsilon_{foam}^{fuel} L_{cell} W_{cell} \delta_{foam}^{fuel}) / \dot{\mathcal{V}}_{in}^{fuel}$<br>The fuel side of the flat-tube SOC consists of cylindrical channels, Ni foam, DL, and FL. The volume of FL is neglected. The detailed structural parameters were provided by Dr.Guan [6].
Planar PEMFC [7] (experiment)	Current	≈ 1.0 [d]	$\tau_m^{fuel} = 0.41$	$\tau_m^{fuel} = (L_{cell} W_{cell} H_{ch}/2 + \varepsilon_{DL}^{fuel} L_{cell} W_{cell} \delta_{DL}^{fuel}) / \dot{\mathcal{V}}_{in}^{fuel}$<br>The total area of the channel is assumed to be the half of the cell area.

[a] The relaxation time is interpreted from figures.
[b] The relaxation time is interpreted from text and figures.
[c] The relaxation time is acquired from the first peak (lowest frequency) in the Distribution of Relaxation Time (DRT) figure.
[d] Cho *et al.* [7] noticed two time delays from the voltage response following a change of current. The first time delay is of the order of 1 s.

Supplementary Figures

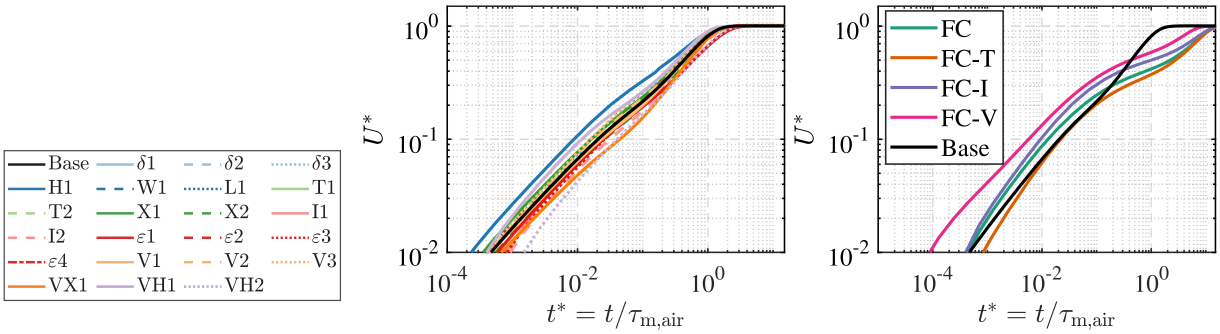

Supplementary Figure 4: This figure serves as an additional component to Fig. 2c,f presented in the main text. When considering $\tau_{\mathrm{m,air}}$ as the time scale, the relaxation time of U^* cannot converge to $1\tau_{\mathrm{m,air}}$.

Nomenclature

Roman symbols

$\dot{\mathcal{H}}$ Enthalpy flow rate, [W]

$\dot{\mathcal{V}}$ Volumetric flow rate, [m^3/s]

$\dot{m}$ Mass flow rate, [kg/s]

$\mathcal{H}$ Enthalpy, [W]

$\mathcal{U}_{\mathrm{R}}$ Utilization rate of reactant

$\mathcal{V}$ Volume, [m^3]

φ_{R} Volume fraction of reactants

$\vec{V}$ Velocity, [m/s]

c_p Specific heat capacity, [J/kg.K]

D Diffusivity, [m^2/s]

d Diameter, [m]

F Faraday constant, 96 485 [C/mol]

H Height, [m]

i Current density, [A/cm^2]

k Thermal conductivity, [W/m.K]

L Length, [m]

m Mass, [kg]

p Pressure, [Pa]

R Ideal gas constant, 8.314 [J/mol.K]

S_{H} Heat source, [W]

S_{h} Volumetric heat source, [W/m^3]

S_{M} Mass source, [kg/s]

S_{m} Volumetric mass source, [kg/m^3.s]

T Temperature, [K]

t Time, [s]

U Voltage, [V]

V_{in} Inlet velocity, [m/s]

W Width, [m]

X_i Mole fraction of species i

Y_i Mass fraction of species i

Greek symbols

α Thermal diffusivity, [m^2/s]

$\bar{\rho}$ Mixture density, [kg/m^3]

δ Thickness, [m]

ρ Density, [kg/m^3]

τ Time constant, [s]

ε Porosity

Subscripts

0 Reference value

ch Fluid channel

eff Effective

f Fluid

h Heat

Int Interconnect

m Mass

P Product

R Reactant

s Solid

Abbreviations

ADL Anode diffusion layer

AFL Anode functional layer

CDL Cathode diffusion layer

CFL Cathode functional layer

DL Diffusion layer

E. Electrolyte

FL Functional layer

SOC Solid oxide cell

SOEC Solid oxide electrolysis cell

SOFC Solid oxide fuel cell

References

[1] Liang, Z., Wang, J., Wang, Y., Ni, M. & Li, M. Transient characteristics of a solid oxide electrolysis cell under different voltage ramps: Transport phenomena behind overshoots. *Energy Conversion and Management* **279**, 116759 (2023).

[2] Bae, Y., Lee, S., Yoon, K. J., Lee, J.-H. & Hong, J. Three-dimensional dynamic modeling and transport analysis of solid oxide fuel cells under electrical load change. *Energy Convers Manage* **165**, 405–418 (2018).

[3] Luo, Y., Shi, Y., Li, W. & Cai, N. Dynamic electro-thermal modeling of co-electrolysis of steam and carbon dioxide in a tubular solid oxide electrolysis cell. *Energy* **89**, 637–647 (2015).

[4] Nerat, M. Modeling and analysis of short-period transient response of a single, planar, anode supported, solid oxide fuel cell during load variations. *Energy* **138**, 728–738 (2017).

[5] Banerjee, A., Wang, Y., Diercks, J. & Deutschmann, O. Hierarchical modeling of solid oxide cells and stacks producing syngas via H2O/CO2 Co-electrolysis for industrial applications. *Appl Energy* **230**, 996–1013 (2018).

[6] Wu, A. *et al.* CO2 utilization by reversible solid oxide cells towards carbon neutralization for long-term energy storage. *Chemical Engineering Journal* **466**, 143275 (2023).

[7] Cho, J., Kim, H.-S. & Min, K. Transient response of a unit proton-exchange membrane fuel cell under various operating conditions. *Journal of Power Sources* **185**, 118–128 (2008).
