# Supplementary material for: Discovering two general characteristic times of transient responses in solid oxide cells
Source: Nat Commun. 2024 May 30;15:4587. doi: 10.1038/s41467-024-48785-1 (PMC11137161; doi:10.1038/s41467-024-48785-1)
Supplement: Supplementary file 3 — Reporting Summary [file 41467_2024_48785_MOESM3_ESM.pdf]

nature portfolio

Corresponding author(s): Mengying Li

Last updated by author(s): Mar 8, 2024

Reporting Summary

Nature Portfolio wishes to improve the reproducibility of the work that we publish. This form provides structure for consistency and transparency in reporting. For further information on Nature Portfolio policies, see our Editorial Policies and the Editorial Policy Checklist.

Statistics

For all statistical analyses, confirm that the following items are present in the figure legend, table legend, main text, or Methods section.

n/a	Confirmed	
☒	☐	The exact sample size (*n*) for each experimental group/condition, given as a discrete number and unit of measurement
☒	☐	A statement on whether measurements were taken from distinct samples or whether the same sample was measured repeatedly
☒	☐	The statistical test(s) used AND whether they are one- or two-sided<br>*Only common tests should be described solely by name; describe more complex techniques in the Methods section.*
☒	☐	A description of all covariates tested
☒	☐	A description of any assumptions or corrections, such as tests of normality and adjustment for multiple comparisons
☒	☐	A full description of the statistical parameters including central tendency (e.g. means) or other basic estimates (e.g. regression coefficient) AND variation (e.g. standard deviation) or associated estimates of uncertainty (e.g. confidence intervals)
☒	☐	For null hypothesis testing, the test statistic (e.g. *F*, *t*, *r*) with confidence intervals, effect sizes, degrees of freedom and *P* value noted<br>*Give P values as exact values whenever suitable.*
☒	☐	For Bayesian analysis, information on the choice of priors and Markov chain Monte Carlo settings
☒	☐	For hierarchical and complex designs, identification of the appropriate level for tests and full reporting of outcomes
☒	☐	Estimates of effect sizes (e.g. Cohen's *d*, Pearson's *r*), indicating how they were calculated

Our web collection on statistics for biologists contains articles on many of the points above.

Software and code

Policy information about availability of computer code

Data collection	The simulation was conducted using a commercial software, Ansys Fluent R18.1.
Data analysis	The data analysis method has been described in the main text, which is simple and straightforward. The code for data analysis is available upon request.

For manuscripts utilizing custom algorithms or software that are central to the research but not yet described in published literature, software must be made available to editors and reviewers. We strongly encourage code deposition in a community repository (e.g. GitHub). See the Nature Portfolio guidelines for submitting code & software for further information.

Data

Policy information about availability of data

All manuscripts must include a data availability statement. This statement should provide the following information, where applicable:

- Accession codes, unique identifiers, or web links for publicly available datasets
- A description of any restrictions on data availability
- For clinical datasets or third party data, please ensure that the statement adheres to our policy

The data that support the plots within this paper are available in the Source data file. Additional data are available from authors upon request.

Research involving human participants, their data, or biological material

Policy information about studies with human participants or human data. See also policy information about sex, gender (identity/presentation), and sexual orientation and race, ethnicity and racism.

Reporting on sex and gender	This is not relevant to our study.
Reporting on race, ethnicity, or other socially relevant groupings	This is not relevant to our study.
Population characteristics	This is not relevant to our study.
Recruitment	This is not relevant to our study.
Ethics oversight	This is not relevant to our study.

Note that full information on the approval of the study protocol must also be provided in the manuscript.

Field-specific reporting

Please select the one below that is the best fit for your research. If you are not sure, read the appropriate sections before making your selection.

☐ Life sciences ☐ Behavioural & social sciences ☒ Ecological, evolutionary & environmental sciences

For a reference copy of the document with all sections, see nature.com/documents/nr-reporting-summary-flat.pdf

Ecological, evolutionary & environmental sciences study design

All studies must disclose on these points even when the disclosure is negative.

Study description	This study investigates the characteristic times of heat and mass transfer within solid oxide cells by numerical simulations. To validate the proposed characteristic times, we conducted an experiment on a solid oxide fuel cell, and collect the current and voltage data for analysis. Besides, relevant data in literature are also used to validate our theory.
Research sample	The solid fuel cell used in the experiment is a commercial product which is different from the one used for the numerical simulation. This experiment shows that the proposed theory is valid for solid oxide cells with different specifications.
Sampling strategy	We applied the Controlled Variables Method in our numerical simulation and experiment. The simulation's sample size was determined to cover all influential factors, based on the number of variables. The experiment focused on a few critical factors due to challenges in manipulating cell structure during experiment. Our theory was tested using our experimental data and literature data. Though no statistical method was used to predetermine the sample size, our approach provides robust support for our theory, ensuring all influential factors were adequately investigated.
Data collection	The numerical simulation data were collected by Zhaojian Liang using a computer. The experimental data were collected by Keda Ren with an electrochemical workstation.
Timing and spatial scale	During the experiment, the current and voltage were recorded with a time interval of 0.1 ms. This sampling rate is sufficient to capture the electrical variations due to mass transfer.
Data exclusions	No data were excluded from the analyses.
Reproducibility	The measurement of current and voltage during the experiment were repeated at least twice. All the measurement attempts are successful.
Randomization	Randomization is not relevant to our study since we do not need to allocate samples into groups.
Blinding	Blinding is not relevant to our study. The data collection was achieve by the computer procedures in commercial softwares.

Did the study involve field work? ☐ Yes ☒ No

Reporting for specific materials, systems and methods

We require information from authors about some types of materials, experimental systems and methods used in many studies. Here, indicate whether each material, system or method listed is relevant to your study. If you are not sure if a list item applies to your research, read the appropriate section before selecting a response.

Materials & experimental systems

n/a	Involved in the study
☒	☐ Antibodies
☒	☐ Eukaryotic cell lines
☒	☐ Palaeontology and archaeology
☒	☐ Animals and other organisms
☒	☐ Clinical data
☒	☐ Dual use research of concern
☒	☐ Plants

Methods

n/a	Involved in the study
☒	☐ ChIP-seq
☒	☐ Flow cytometry
☒	☐ MRI-based neuroimaging

Plants

Seed stocks	This is not relevant to our study.
Novel plant genotypes	This is not relevant to our study.
Authentication	This is not relevant to our study.
